# Experiences on the Utility and Barriers of Telemedicine in Healthcare Delivery in Kenya

**DOI:** 10.1155/2023/1487245

**Published:** 2023-05-03

**Authors:** Simon Onsongo, Charles Kamotho, Tobias F. Rinke de Wit, Kinga Lowrie

**Affiliations:** ^1^Aga Khan Hospital, Kisumu, Box 530-40100, Kisumu, Kenya; ^2^Daktari Africa, Nairobi, Kenya; ^3^Pharmaccess Foundation, Amsterdam, Netherlands; ^4^University of Essex Online, UK

## Abstract

**Introduction:**

Telemedicine is the provision of health services over a distance using information communication technology devices. Telemedicine is emerging as a promising component of healthcare care delivery worldwide, accelerated by the COVID-19 pandemic. This study assessed the factors promoting uptake, barriers, and opportunities for telemedicine among doctors in Kenya. *Methodology*. A semiquantitative, cross-sectional online survey was conducted among doctors in Kenya. During a month, between February and March 2021, 1,200 doctors were approached by email and WhatsApp, of whom 13% responded. *Findings*. A total of 157 interviewees participated in the study. The general usage of telemedicine was 50%. Seventy-three percent of doctors reported using a mix of in-person care and telemedicine. Fifty percent reported using telemedicine to support physician-to-physician consultations. Telemedicine had limited utility as a standalone clinical service. The inadequate information communication technology infrastructure was the most reported barrier to telemedicine, followed by a cultural resistance to using technology to deliver healthcare services. Other notable barriers were the high cost of initial setup limited skills among patients, limited skills among doctors, inadequate funding to support telemedicine services, weak legislative/policy framework, and lack of dedicated time for telemedicine services. The COVID-19 pandemic increased the uptake of telemedicine in Kenya.

**Conclusion:**

The most extensive use of telemedicine in Kenya supports physician-to-physician consultations. There is limited single use of telemedicine in providing direct clinical services to patients. However, telemedicine is regularly used in combination with in-person clinical services, allowing for continuity of clinical services beyond the physical hospital infrastructure. With the widespread adoption of digital technologies in Kenya, especially mobile telephone technologies, the growth opportunities for telemedicine services are immense. Numerous mobile applications will improve access capabilities for both service providers and users and bridge the gaps in care.

## 1. Introduction

Telemedicine refers to using electronic information and communication technologies (ICT) to provide healthcare services over a geographical distance [1]. Telemedicine supports delivery of medical services, medical data exchange, and medical education services, either synchronously or in store-and-forward formats. With rapidly increasing technological innovations, healthcare has a vast array of innovations such as robotics, blockchain technologies, artificial intelligence (AI), the Internet of Medical Things (IOMT), wearable technologies and smart devices, and fast internet connectivity transforming and disrupting the [1, 2]. These technologies offer numerous opportunities to deliver healthcare services through various modalities such as remote patient monitoring (RMP) technologies, virtual consultations, and mobile health (m-Health) [2]. The increase in telemedicine topology, architecture, and platforms provides not only opportunities to increase access but also increase the scope, safety, and quality needs of both users and healthcare providers [3, 4].

Telemedicine narrows the social, economic, and geopolitical barriers between patients and their providers and can support more personalized patient care [5], particularly low- and middle-income countries (LMICs) that are struggling with limited healthcare workforce, brain drain, and limited budget allocations for health [6]. With longer lifespans and an increasing double burden of both infectious and noncommunicable diseases, the strain on the healthcare system will only get worse [7, 8].

Telemedicine can radically increase healthcare access to millions of people around the world if properly utilized [9]. With over 816 million mobile telephony connections by 2019 and an expectation projection of 1.05 billion by the year 2025, the use of mobile devices will not only make it easier to deploy and utilize telemedicine services around the world but also offer personalized medicine [10, 11]. Investing in telemedicine in its various forms in hardware, software, and regulatory elements can complement the existing human resource pool while promoting efficiency and increasing access [11, 12]. Furthermore, coronavirus disease 2019 (COVID-19) has accelerated the adoption and use of telemedicine technologies as part of strategies to stop the pandemic [10, 12–14]. The need to work remotely, preventing the spread of the SARS-CoV-2 virus through social distancing, minimising the minimum number of human contacts, and an increased need to protect high-risk populations have accelerated the adoption of telemedicine around the world [15, 16].

In the post-COVID-19 era, telemedicine is likely to play a more central role in healthcare delivery and constitute an essential pillar for epidemic preparedness and service healthcare delivery over a distance. Although telemedicine is not new to African countries and has wide endorsement among healthcare providers and other healthcare stakeholders, it is not widely utilized [17]. This is due to numerous barriers such as limited access to technology, poor internet infrastructure (particularly in remote and far-to-reach areas), limited healthcare budgets, lack of reimbursement models, high infrastructure costs, limited regulatory oversight, and cultural barriers among users [17].

There is a scarcity of studies on the availability and utilization of telemedicine in healthcare service delivery. As a key alternative route to healthcare care delivery, understanding the determinants of telemedicine adoption, use, and challenges remains important. Some studies reported on the limited use of telemedicine in Kenya [14, 18], but no studies have been carried out to understand the rate of utilization of telemedicine, its determinants, and barriers among physicians in Kenya. This study is aimed at evaluating the rate of use of using telemedicine and factors that affect its utilization in Kenya. Findings from this study will provide useful insights from the perspective of healthcare workers that will help better implementation strategies for telemedicine in Kenya and beyond.

## 2. Methods

### 2.1. Study Methodology

A self-administered, anonymous, cross-sectional web-based study was conducted using an online questionnaire (Qualtrics®, Provo, Utah, United States) through a link shared via email. The software encrypts the data using the Secure Sockets Layer (SSL) and maintains respondents' privacy by masking all respondents' Internet protocol (IP) addresses. Only the principal investigator could access the collected data through a password-protected portal. The study population was Kenyan physicians who are members of the Kenya Medical Association (KMA) in 47 counties in Kenya.

### 2.2. Inclusion and Exclusion Criteria

#### 2.2.1. Inclusion Criteria

- Providing informed consent
- Membership in the Kenya Medical Association
- Involvement in the clinical care of patients

#### 2.2.2. Exclusion Criteria

- Nurses and allied health care staff
- Doctors not residing in Kenya at the time of the survey

### 2.3. Sample Size Calculation

Using the teleradiology rate of 26% reported in Uganda [19] and assuming an 80% confidence level with a finite population correction of *n* = 1,200 (population of doctor members of KMA) and a nonresponse rate of 30%, we obtained a total sample size of 150 [20].

### 2.4. Study Tools and Data Management

The questionnaire was adapted from Vidal-Alaball et al. [21]. The final version was piloted among ten doctors—to assess user access, feedback, acceptance, and overall flow of questions. An email was sent to each study participant containing survey instructions and an active link to the online survey. The data collected were stored in real time on a secure online cloud storage platform with password-protected access to the principal investigator. Participant privacy was maintained during the study since the data collected was anonymized as participant names, Internet protocol (IP) addresses, and email addresses were not captured.

### 2.5. Research and Ethical Considerations

Before starting data collection, all research and ethical approvals were obtained from the Research and Ethics Review Committee of the Jaramogi Oginga Odinga Teaching & Referral Committee Hospital (license number IERC/JOOTRH/369/2020) and the National Commission for Science, Technology, and Innovation (NACOSTI), license number NACOSTI/P/21/8668. Informed consent was obtained from all study participants at the start of the survey.

## 3. Results

**Figure 1:**
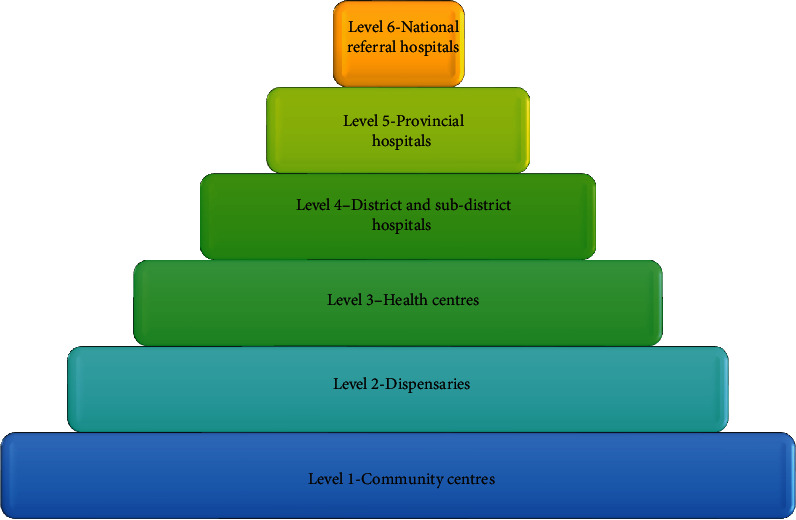
The multilevel healthcare system in Kenya consists of different levels of care, including community health services, dispensaries, health centres, and hospitals. At the primary level, community health services and dispensaries provide basic healthcare services to the population. Hospitals at the secondary and tertiary levels provide more specialized medical services with advancement in specialty with each increasing level.

**Figure 2:**
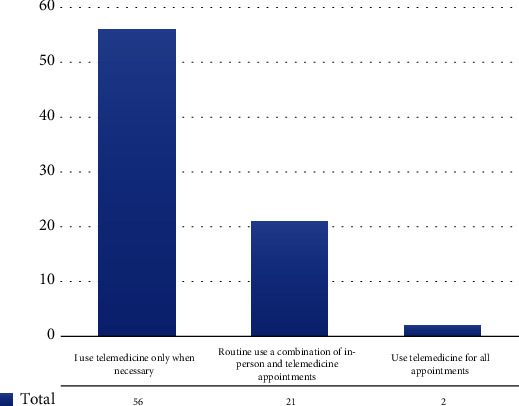
Physician patterns of telemedicine use in their routine practice.

The study took place from February 25 to March 8th, 2021, recruiting 157 fully completed surveys included in the analysis. The demographic characteristics of the study population are shown in Table 1. Survey responses were obtained from Kenya's 25/47 (53%) counties. Two counties (Nairobi and Kisumu) represented the highest number of participants (59%). Most of the study participants (84%) were between 30 and 50 years of age, with an equal representation from both genders. Responses were obtained from healthcare facilities (43.3%), private doctors (33.8%), and academic institutions (17.2%). The responses from public health facilities were from the district and national referral facilities from level 4 to level 6, as highlighted in Figure 1. Half of the study participants reported using telemedicine services in the recent past. Most study participants (83.5%) reported having used telemedicine for less than five years (Table 2). The percentage of hands-on usage time was less than 25% in 83.5% of all the respondents. One study participant reported using telemedicine services over 75% of the time for healthcare delivery (Figure 2). Twenty-six percent (26%)of physicians reported a mixed use of telemedicine to support the delivery of health services. These included supporting routine diagnosis and treatment of patients, emergency care services, physician-to-physician consults, and psychotherapy/behavioural services (Table 2). Fifty-one percent of physicians reported using telemedicine to support physician-to-physician consultations. The most predominant form of telemedicine used was store-and-forward, as shown in Table 2. Inadequate informational communication technology (ICT) infrastructure was the most cited barrier (80%), followed by cultural resistance by users (59.5%), high cost of initial setup (53.2%), lack of skills among patients (51.9%), limited skills among doctors (39.2%), inadequate budgetary allocation (35%), and weak legislative framework (32.9%), as summarized in Table 2. Suggestions to improve the uptake of telemedicine included inclusion in insurance reimbursement packages (69%), increased networking among telemedicine users, adoption of better ICT support, and greater clarification around legal, security, and regulatory issues on the use of telemedicine (Table 2). Some respondents suggested that a pricing model that adequately compensates doctors and promotes access among users (patients) would support increased adoption and make these services more sustainable.

The COVID-19 pandemic stimulated the adoption of telemedicine services in Kenya, as noted by 83% of the respondents. Seventy-four percent of respondents estimated the impact of SARS-CoV-2 on telemedicine to be a modest range of between 10 and 49%. A high proportion of the respondents (91%) confirmed the continued use of telemedicine into the future. Of the participants without access to telemedicine services, only 20% had plans to introduce the services in the short term (next 12-24 months). Almost all respondents (95%) without access to telemedicine services confirmed willingness to utilize it, once available.

## 4. Discussion

The 21st century has seen an unprecedented increase in the adoption and use of technology in many sectors, including healthcare. Application of information and communication technologies (ICT) in healthcare can result in increased access, better efficiency, convenience, greater quality, and more personalized services [22, 23]. LMICs have serious inequities in access to healthcare [24]. With inadequate human resources, inadequate budgetary support, and poor healthcare management, LMICs will require innovative and radical approaches to improve access to healthcare services [24]. With a youthful and rapidly growing population, a brain drain of the healthcare workforce, and a double burden of infectious and noncommunicable diseases, the healthcare demands are grossly unmatched [25]. Telemedicine provides opportunities to increase access to needy and far-to-reach communities by leveraging the use of various digital technologies to bypass traditional access barriers.

This study assessed the rate and positive and negative determinants of telemedicine uptake among doctors in Kenya. Responses were received from 157 doctors working in different parts of the country, though there were limited responses from lower-level (level 1 to level 3) facilities in Kenya (as shown in Figure 1), due to a limited number of doctors deployed in those centres. In Kenya, community and primary healthcare centres ranging from levels 1 to 3 are manned by nurses, community health workers, and clinical officers. A low response rate of 13% points to the difficulty of conducting online surveys where participants may not have intrinsic motivation or incentive to participate in the study. Though the study response rate was low, the study respondents were received from across 24 out of 47 counties in Kenya.

The rate of telemedicine usage among physicians in Kenya in this survey was 50%. The highest utility of telemedicine was in supporting physician-to-physician consults and education with limited use in supporting direct patient care in Kenya. These physician-to-physician consults or e-consults allow for quick specialist advice to be shared with nonspecialist colleagues serving in remote or distant locations allowing for quick and easy information sharing especially for rare, complex, and unusual clinical cases. Teleconsultations between geographically separated physicians may provide opportunities to improve healthcare delivery by quickening the referral process, change in diagnosis, or treatment plan for the patient [26]. With the deployment of newer technologies such as artificial intelligence technologies (AI), the speed of detection of decision-making could rapidly be increased where data or images are utilized for diagnostic support [27]. A study in Abu Dhabi in the Middle East by Alhajri et al. [28] showed that the physician's confidence in the management of acute conditions increased through virtual video consultations leading to better patient care. Other authors [29, 30] have found similar favourable findings for e-consults. Similarly, Lapadula et al. [31] noted a high level of satisfaction among patients and neonatologists who were involved in neonatal and prenatal virtual consults during the COVID-19 pandemic. This highlights the key role that e-consultations can increase access to and reducing waiting times for specialist care. As the number of tools for digital increases, and collaboration increases, the value of high accessibility will become and becomes evident, perhaps translating to better care and improved patient outcomes.

In contrast, minimal patient-to-doctor utilization was reported as shown in Table 2 showing low utilization of telemedicine in the delivery of clinical care services. These findings are not unusual and have been reported in other studies throughout the continent [32–35], highlighting significant barriers that limit access to telemedicine that can support direct clinical care. The limited availability of information and communication technology (ICT) capacity has been cited by many as the main barrier [34]. Factors such as unreliable electricity supply, lack of reliable internet, unavailability of inadequate infrastructure, lack of telemedicine-ready equipment, and lack of adequate technical support may make telemedicine services difficult to support [36]. In their meta-analysis, Dodoo et al. [37] reported the availability of a wide range of telemedicine technologies across the African continent but noted the limited utility during the COVID-19 pandemic. The authors noted that in Kenya, technological barriers mainly were related to inadequate and inappropriate ICT infrastructure. This finding is consistent with our study findings. Limited access to digital-enabled devices, lack or limited internet connectivity, and high internet costs may limit deserving rural communities from accessing available telemedicine services [38, 39]. With the increasing connectivity of smartphones in Kenya and other LMICs [40], the opportunity to take advantage of the use of mobile health applications provides a radical and innovative approach to bridge the gap.

The limited ICT capacity could partially explain a moderate increase in the adoption of telemedicine services (between 10 and 49%) reported in this study despite the considerable need during the ongoing coronavirus pandemic. The emergency of a global pandemic caused by SARS-CoV2 and the need to reduce viral spread, protect vulnerable populations, and provide continuity of medical care provided an opportunity to scale up alternative healthcare delivery models. To make our healthcare system ready for future pandemics and other emerging disease outbreaks, a robust digital ecosystem that supports telemedicine activities is desirable. Apart from physical ICT infrastructure and fast internet connectivity, a policy and regulatory framework that encourage utilization of telemedicine are required. According to Chitungo et al. [35], additional multiple interventions are required to increase the utilization of telemedicine. These may include workload redesign for healthcare workers to include telemedicine components, roll-out of mass trainings, incorporation of local multilanguage support, and partnership with vendors and other private partners to support a functional telemedicine ecosystem in Africa. Healthcare leaders and policymakers must prioritize the allocation of healthcare budgets to better support the telemedicine and better infrastructure and tap into the benefits of telemedicine in healthcare delivery. The high costs of internet connectivity and data bundles should also be considered so that rural poor and remote locations can access health services.

Limited insurance coverage and lack of payment models remain an important obstacle in this study, as noted in Table 2. Compensation for telemedicine services is critical for optimal service uptake [41]. Governments, insurance companies, and other healthcare providers around the world are developing various compensation models to enhance the optimal uptake of telemedicine. In the recent past, the Centers for Medicare & Medicaid Services (CMS) has widened the scope of services covered through the generation of new billing codes [42], rapidly increasing the coverage around the United States of America and signaling a positive shift that increases access to many. The Australian government not only increased the scope of services covered but also invested AUD 100 million (USD 68 million) to support telemedicine services across a wide range of at-risk populations [43]. With limited healthcare budget allocations and increasing healthcare costs, healthcare providers will be seeking telemedicine and other alternative healthcare models that offer affordability, access, efficiency, and quality. As we move away from the traditional restrictive payment models, we must enhance payment parity and payment equity to encourage utilization of telemedicine services across various specialties [44].

Finally, we must also overcome cultural resistance against telemedicine. Doctors consider telemedicine to be vastly different from traditional clinical delivery methods; hence, it is considered disruptive and complex and, in most cases, requires physicians to learn a new set of skills [45] and redesign the current models of care. Loss of personal connection may make some physicians to shy away from telemedicine. Limited availability of telemedicine services, the need to learn new skills, and lack of awareness on the benefits of telemedicine may aggravate cultural resistance to telemedicine among users. To enhance the uptake and diffusion of new telemedicine technologies, both physicians and their patients must overcome cognitive, emotional, and contextual concerns that may hinder uptake [46]. Changing malleable negative perceptions about the use of technology in healthcare delivery will lead to better and sustainable adoption. Both physicians and patients should be supported with telemedicine technologies that are useful in meeting their objectives, are accessible and easy to use, and are consistent with the technology acceptance model (TAM) [47]. The lack of telemedicine curricula in most physician programs makes breaking free from cultural resistance even more challenging.

Although there are numerous barriers to the use of telemedicine in Kenya, study participants show great enthusiasm and willingness to continue using telemedicine in the foreseeable future. Increased enthusiasm to use telemedicine or to use it when it becomes available is commendable and signals the likelihood of increased adoption as we move into the post-COVID-19 era. Those without telemedicine also showed a willingness to use it once it is available, suggesting the likelihood of high adoption rates. Providers also suggested areas where improvement is needed to better support telemedicine. Focus on these areas will lead to better utilization of telemedicine in the delivery of healthcare services.

## 5. Conclusion

Technology plays a pivotal role in the delivery of healthcare services around the world, with telemedicine becoming a vital component of the healthcare system. Telemedicine offers an opportunity to scale up healthcare services to those in need, particularly those in remote, distant, or not easily accessible locations. In this study, we demonstrate a high rate of telemedicine awareness and utilization among doctors in Kenya. The current utilization in Kenya is to support doctor-to-doctor consultations and provide education with minimal utilization in actual healthcare delivery. Numerous barriers to the use of telemedicine are noted.

We showed that the coronavirus pandemic had a modest increase in the utilization of telemedicine for healthcare services delivery during the pandemic period probably due to limited infrastructure to scale up. Indeed, to maximize the utility of telemedicine usage in Africa and other developing countries, significant improvements are needed in multiple areas cutting across regulatory, infrastructural, legal, and financial, to better support telemedicine services and improve healthcare delivery. The inclusion of telemedicine curricula in the training programs of health workers may also promote awareness and uptake of telemedicine in Kenya.

## 6. Study Limitations

While this study provides important information on the telemedicine practices of physicians in Kenya, it is subject to notable limitations. As a cross-sectional study, it is unable to establish causal relationships between variables. Additionally, the findings are not indicative of potential changes in behaviour over time. The study was conducted solely with physicians, and as such, the findings may not be generalizable to the entire healthcare workforce in Kenya. Furthermore, the study's response rate was heavily biased towards physicians residing in major cities, with nearly half of all respondents hailing from just two major towns. This presents a potential source of bias, as urban and rural telemedicine experiences among physicians may differ significantly. Finally, while responses were collected from 24 out of the 47 counties, the low response numbers in some counties further impact the generalizability and bias of the findings.

## Figures and Tables

**Table 1:** Demographic characteristics of the study population.

Variables	Telemedicine		Total	Pearson's chi-square	*P* value
	Without telemedicine	With telemedicine			
Age				0.4006	0.818
Below 30 years	8 (57.1%)	6 (42.9%)	14 (100%)		
Between 30 and 50 years	65 (49.2%)	67 (51.8%)	132 (100%)		
More than 50 years	5 (45.5%)	6 (55.5%)	11 (100%)		
Total	78 (49.7%)	79 (50.3%)	157 (100%)		
Gender				1.0755	0.300
Female	36 (45.6%)	43 (54.4%)	79 (100%)		
Male	42 (53.9%)	36 (46.1%)	78 (100%)		
Total	78 (49.7%)	79 (50.3%)	157 (100%)		
Education level				2.7232	0.605
Degree	20 (52.6%)	18 (47.4%)	38 (100%)		
Diploma	0 (0.00%)	2 (100%)	2 (100%)		
Fellowship	7 (43.8%)	9 (56.2%)	16 (100%)		
Masters	50 (51.0%)	48 (49.0%)	98 (100%)		
PhD	1 (33.3%)	2 (66.7%)	3 (100%)		
Total	78 (49.7%)	79 (50.3%)	157 (100%)		
Specialities				10.9602	0.532
Anaesthesiology	3 (100%)	0 (0%)	3 (100%)		
Emergency medicine	1 (100%)	0 (0%)	1 (100%)		
Family medicine	0 (0%)	1 (10%)	1 (100%)		
General practitioner	3 (50%)	3 (50%)	6 (100%)		
General surgery	2 (67.7%)	1 (32.3%)	3 (100%)		
Haematology	0 (0%)	1 (100%)	1 (100%)		
Internal medicine	4 (57.1%)	3 (42.9%)	7 (100%)		
Obstetrics and Gynaecology	7 (63.6%)	4 (36.4%)	11 (100%)		
Paediatrics	3 (75%)	1 (25%)	4 (100%)		
Pathology	12 (52%)	13 (48%)	25 (100%)		
Psychiatry	1 (50%)	1 (50%)	2 (100%)		
Radiology	1 (20%)	4 (80%)	5 (100%)		
Others	12 (41.4%)	17 (58.6%)	29 (100%)		
Total	50	48	98 (100%)		
Institution				6.7468	0.080
Individual or group	4 (44.4%)	5 (5.6%)	9 (100%)		
Private sector	19 (35.9%)	34 (64.1%)	53 (100%)		
Public healthcare	39 (57.4%)	29 (42.6%)	68 (100%)		
University/college	16 (59.3%)	11 (40.7%)	27 (100%)		
Total	78 (49.7%)	79 (50.3%)	157 (100%)		

**Table 2:** The different forms, barriers, and opportunities related to the adoption of telemedicine among doctors in Kenya, as presented in the subthemes.

	Frequency	Percentage
*Subtheme 1: patterns of usage of telemedicine services*		
Do you have any telemedicine services in your institution?		
Yes	79	50.3%
For how long have you used telemedicine in delivering patient care?		
Less than 5 years	66	83.5%
Between 5 and 10 years	9	11.4%
More than 10 years	4	5.1%
What percentage of time do you spend providing care to patients via telemedicine?		
Less than 25%	65	82.3%
25-49%	10	12.7%
50-74%	3	3.8%
More than 75%	1	1.3%
*Subtheme 2: utilization of telemedicine*		
For diagnosis and treatment of patients	39	51%
Chronic disease management	32	42%
Emergency care of patients	12	16%
The physician-to-physician consults	39	51%
Behavioural services	4	5%
Critical care services	4	5%
Others	18	23%
Total	148	100%
*Subtheme 3: forms of telemedicine in use*		
Real-time telemedicine—e.g., use of live video consultations	28	37%
Remote patient monitoring (monitoring of patients outside of conventional clinical settings)	16	21%
Archived and uploaded images or text transmission to be reviewed by the doctor	54	71%
Total	98	100%
*Subtheme 4: barriers to telemedicine*		
Inadequate ICT infrastructures	64	80%
Cultural resistance by users and providers	47	60%
Costly to set up and manage	42	53%
A lack of skills among patients	41	52%
Privacy concerns	32	41%
A lack of skills among care providers	31	39%
Inadequate funding	28	35%
An inadequate legislative framework	26	33%
Others	4	5%
*Subtheme 5: ways to improve uptake of telemedicine*		
Approve the use of insurance payments for telemedicine services	54	69.2%
Networking with more telemedicine users	55	69.6%
Improve audio/video quality	46	58.2%
Improve technical issues and bugs	40	50.6%
Address legal and regulatory concerns	48	60.8%
Address security concerns	39	49.4%
Make it easier to use	39	49.4%
Others	1	1.3%
*Subtheme 6: impact of COVID-19 on the uptake of telemedicine*		
Did the 2020 coronavirus pandemic increase the uptake of telemedicine services in your facility?		
No	8	10.1%
Not sure	5	6.3%
Yes	66	83.5%
By what margin did your use of telemedicine services increase during the 2020 coronavirus pandemic?		
10-49%	48	73.8%
50-99%	17	26.2%
*Subtheme 7: potential to use telemedicine once available*		
If given the opportunity, will you consider using telemedicine to provide healthcare services to your patients?		
Not sure	4	5.1%
Yes	74	94.9%

## Data Availability

The study data that support the findings of this study are available upon request to the corresponding author.
